# Heterogenous Distribution of *MTHFR* Gene Variants among Mestizos and Diverse Amerindian Groups from Mexico

**DOI:** 10.1371/journal.pone.0163248

**Published:** 2016-09-20

**Authors:** Cecilia Contreras-Cubas, Beatríz E. Sánchez-Hernández, Humberto García-Ortiz, Angélica Martínez-Hernández, Francisco Barajas-Olmos, Miguel Cid, Elvia C. Mendoza-Caamal, Federico Centeno-Cruz, Gabriela Ortiz-Cruz, José Concepción Jiménez-López, Emilio J. Córdova, Eva Gabriela Salas-Bautista, Yolanda Saldaña-Alvarez, Juan Carlos Fernández-López, Osvaldo M. Mutchinick, Lorena Orozco

**Affiliations:** 1 Inmunogenomics and Metabolic Disease Laboratory, Instituto Nacional de Medicina Genómica, SS, Mexico City, Mexico; 2 Genetic Department, Instituto Nacional de Ciencias Médicas y Nutrición Salvador Zubirán, SS, Mexico City, Mexico; 3 Physic Anthropology Direction, Instituto Nacional de Antropología e Historia, Museo Nacional de Antropología, Mexico City, Mexico; 4 Computational Genomics, Instituto Nacional de Medicina Genómica, SS, Mexico City, Mexico; Centre Hospitalier Universitaire Sainte-Justine, CANADA

## Abstract

Methylenetetrahydrofolate reductase (MTHFR) is a key enzyme in folate metabolism. Folate deficiency has been related to several conditions, including neural tube defects (NTDs) and cardiovascular diseases. Hence, *MTHFR* genetic variants have been studied worldwide, particularly the C677T and A1298C. We genotyped the C677T and A1298C *MTHFR* polymorphisms in Mexican Amerindians (MAs), from the largest sample included in a genetic study (n = 2026, from 62 ethnic groups), and in a geographically-matched Mexican Mestizo population (MEZ, n = 638). The 677T allele was most frequent in Mexican individuals, particularly in MAs. The frequency of this allele in both MAs and MEZs was clearly enriched in the South region of the country, followed by the Central East and South East regions. In contrast, the frequency of the 1298C risk allele in Mexicans was one of the lowest in the world. Both in MAs and MEZs the variants 677T and 1298C displayed opposite allele frequency gradients from southern to northern Mexico. Our findings suggest that in Mestizos the 677T allele was derived from Amerindians while the 1298C allele was a European contribution. Some subgroups showed an allele frequency distribution that highlighted their genetic diversity. Notably, the distribution of the frequency of the 677T allele was consistent with that of the high incidence of NTDs reported in MEZ.

## Introduction

Methylenetetrahydrofolate reductase (MTHFR) is a key enzyme in the folate metabolic pathway. This enzyme catalyzes the reduction of 5,10-methylenetetrahydrofolate to 5-methyltetrahydrofolate, which provides a methyl group for the conversion of homocysteine to methionine [1]. Among the most common genetic variants in the *MTHFR* gene are the non-synonymous polymorphisms, C677T and A1298C. The C677T polymorphism changes an alanine to valine (A222V), and the A1298C variant changes a glutamic acid to alanine (E429A). Both polymorphisms have been associated with reduced enzyme activity, particularly the C677T polymorphism, which imparts thermolability to the enzyme [2, 3]. Worldwide studies of these variants have shown that the Mexican Mestizo population has the highest frequency of the 677T allele (44–61%), and the lowest frequency of the 1298C allele (7–11.7%) [4–8]. In particular, the C677T polymorphism has been implicated in several diseases, including neural tube defects (NTDs), cardiovascular diseases, hypertension and various types of cancer, among others [4, 9–14]. Remarkably, NTDs are of the most prevalent congenital malformations in Mexico; the prevalence that has been estimated is at least 1 in 250 conceptions that reach 20 weeks of pregnancy [4]. Furthermore, between 1998 and 2006, NTDs represented 39.63–56.91% of deaths by nervous system defects [15].

Previous studies have suggested that the Amerindian genome has largely contributed to the presence of the 677T allele in the Mexican-Mestizo population [7, 8, 13, 16]. Most of Mexicans are a mixed population derived mainly from Amerindian, European, and African ancestries, whose proportion vary largely across the country, with a clear increasing Amerindian ancestry gradient from North to South [17, 18]. Additionally, 14.9% of the Mexican population is indigenous, distributed in 68 different ethnic groups [19, 20]. Despite the relevance of the *MTHFR* C677T variant in populations with an Amerindian origin, genetic studies have explored only about 10% of the ethnic groups recognized by the Mexican Indigenous Languages Institute (INALI) [7, 8, 13, 16, 20]. Moreover, very few studies have examined the A1298C variant in the Mexican population, even in the Mestizo population.

Here, we investigated the allele and genotype frequencies of the *MTHFR* C677T and A1298C variants and their geographic distributions in the largest cohort of Mexican Amerindians (MAs) included in a genetic study. We also included a sample of Mexican Mestizo (MEZ) individuals geographically-matched.

## Materials and Methods

### Ethics Statement

This study was conducted in accordance with the Declaration of Helsinki. Local ethics and research committees of Instituto Nacional de Medicina Genómica approved this study. All MA participants provided informed written consent, and parents provided consent for their MEZ newborn infant participation. When necessary, the leader or bilingual members of the community translated the informed consent into the native language and analphabetic individuals signed with their fingerprints.

### Study Population

This study included 2026 Mexican AM individuals from 62 different ethnic groups, which belong to the Metabolic Analysis in an Indigenous Sample (MAIS) cohort. Inclusion criteria were that the individuals identified themselves as indigenous, that their parents and grandparents spoke the same language, and that they were born in the same area as their parents and grandparents. We also included 638 MEZ newborn infants, which were matched with MA individuals by geographic region.

Since there is a high genetic heterogeneity in the Mexican population and a clear European ancestry gradient North-South in MEZ [18, 21, 22, 23], both MA and MEZ populations were sorted into five geographic regions, modified from those regions described in the Mexican Official National Journal and taking into consideration previously published genetic data: North (N), Central East (CE), Central West (CW), South (S), and South East (SE)[18].

### Genotyping

Genomic DNA was extracted from whole peripheral blood samples with the QIAmp DNA Blood Maxi kit (Qiagen Systems, Inc., Valencia CA), according to the manufacturer′s protocol. The C677T (rs1801133) and A1298C (rs1801131) SNPs in *MTHFR* were genotyped by allelic discrimination with the TaqMan^®^ SNP genotyping assay on a 7900HT Fast Real-Time PCR System (Applied Biosystems, Life Technologies CA, USA). The call rate exceeded 96% for both SNPs, and no discordant genotypes were observed in the 10% duplicate samples that served as a quality control. Genotypes were confirmed by randomly sequencing 10% of the samples, with an automated ABI PRISM 310 Genetic Analyzer (Applied Biosystems, Life Thechonolgies, CA, USA), which showed 100% reproducibility. Ancestry was confirmed in a random sample of 1304 AM individuals with the 6.0 SNP array (Affymetrix, Santa Clara, CA) or GoldenGate genotyping assay (Illumina, San Diego, CA). This last SNP array contained 96 ancestry markers, validated in other studies [24].

### Statistical Analysis

To estimate the allele and genotype frequencies, we considered that several Mexican indigenous groups have a small effective population size and few possibilities of outbreeding, then, we included those ethnic groups with a sample size of at least 10 individuals. Thus, to estimate allele frequencies we considered only 31 ethnic groups, including 5 Nahuatl groups from different geographic regions: Mexico City (CDMX), Mexico (self-recognized as “Mexicano”), Morelos, Puebla, and San Luis Potosí States. Genotype and allele frequency comparisons were performed with Chi-square analyses, with PLINK software, v1.07 [25]. Significant differences were defined as *P*<0.05. For further allele frequency comparisons between MAs and MEZs by geographical regions, we excluded Seris (N), Pames (CE), Huaves (S), and Tojolabales (SE) because they fulfilled at least two of the following three criteria: a) in previous studies, they were reported as genetically divergent from all other ethnic groups [18], b) in this study, their 677T and 1298C allele frequencies were significantly different from all the MAs inhabiting the same geographical region; and c) according to historical data, they have not contributed to the genetic structure of the MEZs [26–28]. Allelic distribution was represented on maps taken from the National Commission of Knowledge and Use of Biodiversity (CONABIO) [29]. Maps were modified according to the allelic frequencies. Haplotype frequencies and linkage disequilibrium (LD) (r^2^) were determined with the Haploview program [30].

## Results

### Genotype and allele frequencies of *MTHFR* C677T and A1298C polymorphisms in MA and MEZ populations

An ancestry analysis displayed an average Amerindian ancestry of 95 ± 5.7% in the MA sample. The genotype distribution for the two *MTHFR* SNPs evaluated was in Hardy–Weinberg equilibrium, in both MA and MEZ populations. To gain global insight into the frequency of the C677T and A1298C polymorphisms in the Mexican population, we compared the genotype and allele frequencies observed in the MA (n = 2026) and MEZ (n = 638) populations with those reported for continental populations, such as Africans (AFR), Asians (CHB), and Europeans (EUR), as well as the Mexican Ancestry individuals from Los Angeles, CA, USA (MXL) from the 1000 genome database [31]. The major European contribution to the Mexican gene pool is from Spain; therefore, we also compared frequencies to the Iberian population (IBS) (Table 1). The MA population had the highest frequencies in the world for the 677T risk allele and the TT genotype (65% and 44.5%, respectively), followed by the MEZ (52% and 26%, respectively) and MXL (47% and 20.3%, respectively) samples. The frequencies observed in MAs were significantly different from those observed in the MEZ population from this study and from those observed in populations reported in the 1000 genomes database (S1 Table) [31]. Conversely, the MA population had the lowest frequencies in the world for the 1298C risk allele and CC genotype (6% and 0.4%, respectively). Low frequencies for the 1298C risk allele and CC genotype were also found in the MEZ population included in the present study (14% and 2%, respectively) and in that reported in the MXL population (16% and 1.8%, respectively) (Table 1) [31].

**Table 1:** Genotype and minor allele frequencies of *MTHFR* C677T and A1298C variants in different populations, including those in the present study, MAs and MEZs.

	C677T				A1298C			
	C677T_Genotype			Allele Frequency	A1298C_Genotype			Allele Frequency
Population(N:C677T/A1298C)	CC_ Count(%)	CT_Count(%)	TT_Count(%)	T%	AA_Count(%)	AC_Count (%)	CC Count (%)	C %
Mexican_Amerindians(MA)*(1942/2026)	293(15%)	786(40.5%)	863(44.5%)	65	1797(88.6%)	223(11%)	6(0.4%)	6
Mexican_Mestizo(MEZ)*(607/537)	139(23%)	309(51%)	159(26%)	52	399(74%)	130(24%)	8(2%)	14
Mexicans_LA(MXL)**(64)	17(26.5%)	34(53.2%)	13(20.3%)	47	44(68.5%)	19(29.7%)	1(1.8%)	16
Iberians(IBS)**(107)	30(28%)	59(55.2%)	18(16.8%)	44	55(51.4%)	46(43%)	6(5.6%)	27
Europeans(EUR)**(503)	204 (40.5%)	231(46%)	68(13.5%)	36	239(47.5%)	213(42.3%)	51(10.2%)	31
Asians(CHB)**(504)	259(51.4%)	192(38%)	53(10.6%)	30	302(60%)	183(36.3%)	19(3.7%)	22
Africans(AFR)**(706/661)	594(84%)	105(15%)	7(1%)	9	478(72.3%)	166(25.1%)	17(2.6%)	15

*Present Study

**1000 Genomes Database

**Fig 1:**
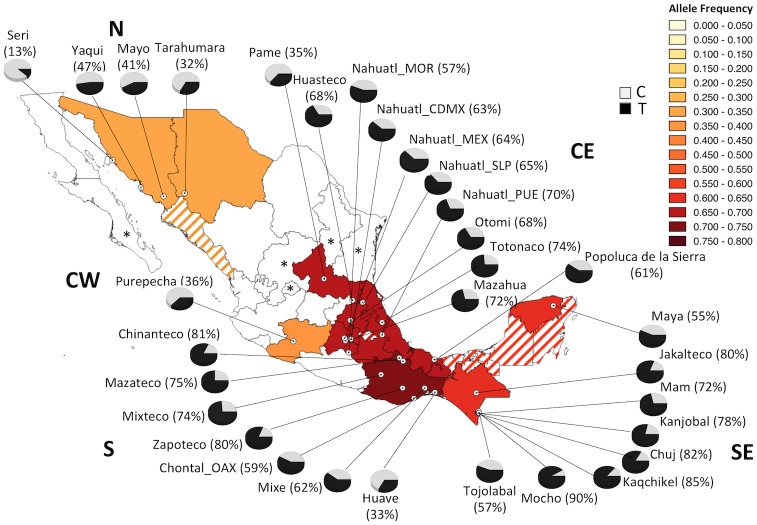
Geographic distribution of allele frequencies for the C677T polymorphism in the MA population. CDMX, Mexico City; MEX, Mexico State; MOR, Morelos; OAX, Oaxaca; PUE, Puebla; SLP, San Luis Potosí. Striped States were not sampled because they are inhabited by neighboring indigenous included in this study. *States without indigenous population [32].

**Fig 2:**
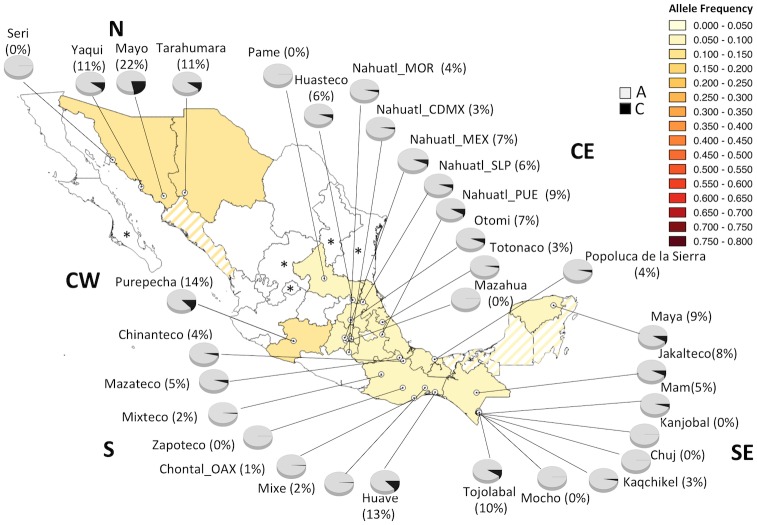
Geographic distribution of allelic frequencies for the A1298C polymorphism in the MA population. CDMX, Mexico City; MEX, Mexico State; MOR, Morelos; OAX, Oaxaca; PUE, Puebla; SLP, San Luis Potosí. Striped States were not sampled because they are inhabited by neighboring indigenous included in this study. *States without indigenous population [32].

We were able to evaluate the C677T and A1298C allele and genotype frequencies in 31 different MA groups, where more than 10 individuals were recruited (Table 2). The 31 MA groups and the MEZ populations were sorted in five major geographic regions in Mexico (N, CW, CE, S and SE). In the MA ethnic groups, this analysis showed a high heterogeneity of the allele and genotype frequencies for both polymorphisms, which followed a geographic gradient across Mexico. Allelic and genotype frequencies of both *MTHFR* polymorphisms in Seri, Huave, Pame, and Tojolabal ethnic groups were significantly different compared with their neighbors in the same region. The five Nahuatl groups from different geographic regions showed different genotype and allele frequencies (Table 2; Figs 1 and 2).

**Table 2:** Geographic distribution of genotype frequencies for *MTHFR* C677T and A1298C polymorphisms among 31 MA ethnic groups and MEZs.

POPULATIONS		GENOTYPIC FREQUENCIES											
		C667T						A1298C					
GEOGRAFICAL REGION	ETHNIC GROUP (N:C677T/A1298C)	CC		CT		TT		AA		AC		CC	
		Count	Freq.	Count	Freq.	Count	Freq.	Count	Freq.	Count	Freq.	Count	Freq.
**NORTH (N)**	**MAYO (28/29)**	10	0.36	13	0.46	5	0.18	17	0.59	11	0.38	1	0.03
	**SERI (19/19)**	14	0.74	5	0.26	0	0.00	19	1.00	0	0.00	0	0.00
	**TARAHUMARA (88/92)**	40	0.46	39	0.44	9	0.10	71	0.77	21	0.23	0	0.00
	**YAQUI (37/37)**	9	0.24	21	0.57	7	0.19	29	0.78	8	0.22	0	0.00
	**TOTAL AMERINDIANS (172/177)**	73	0.43	78	0.45	21	0.12	136	0.77	40	0.22	1	0.01
	**MESTIZO (85/83)**	36	0.42	36	0.42	13	0.15	48	0.58	33	0.40	2	0.02
**CENTRAL WEST (CW)**	**PUREPECHA (14/14)**	6	0.43	6	0.43	2	0.14	10	0.71	4	0.29	0	0.00
	**TOTAL AMERINDIANS (14/14)**	6	0.43	6	0.43	2	0.14	10	0.71	4	0.29	0	0.00
	**MESTIZO (64/65)**	25	0.39	27	0.42	12	0.19	36	0.55	28	0.43	1	0.02
**CENTRAL EAST (CE)**	**HUASTECO(79/78)**	9	0.11	32	0.41	38	0.48	69	0.88	9	0.12	0	0.00
	**MAZAHUA (10/10)**	0	0.00	6	0.60	4	0.40	10	1.00	0	0.00	0	0.00
	**NAHUATL CDMX (52/52)**	7	0.13	25	0.48	20	0.38	49	0.94	3	0.06	0	0.00
	**NAHUATL MEX (22/22)**	3	0.14	10	0.45	9	0.41	19	0.86	3	0.14	0	0.00
	**NAHUATL MOR (44/45)**	6	0.14	26	0.59	12	0.27	41	0.91	4	0.09	0	0.00
	**NAHUATL PUE (52/52)**	3	0.06	25	0.48	24	0.46	44	0.85	7	0.13	1	0.02
	**NAHUATL SLP (44/44)**	4	0.09	23	0.52	17	0.39	39	0.89	5	0.11	0	0.00
	**OTOMI (220/219)**	29	0.13	84	0.38	107	0.49	188	0.86	30	0.14	1	0.00
	**PAME (10/10)**	3	0.30	7	0.70	0	0.00	10	1.00	0	0.00	0	0.00
	**POPOLUCA DE LA SIERRA (36/36)**	5	0.14	18	0.50	13	0.36	34	0.94	1	0.03	1	0.03
	**TOTONACO (97/95)**	8	0.08	34	0.35	55	0.57	89	0.94	6	0.06	0	0.00
	**TOTAL AMERINDIANS (666/663)**	77	0.12	290	0.44	299	0.45	592	0.89	68	0.10	3	0.01
	**MESTIZO (324/263)**	56	0.17	174	0.54	94	0.29	208	0.79	50	0.19	5	0.02
**SOUTH (S)**	**CHINANTECO (81/83)**	4	0.05	22	0.27	55	0.68	77	0.93	6	0.07	0	0.00
	**CHONTAL DE OAXACA (44/44)**	10	0.23	16	0.36	18	0.41	43	0.98	1	0.02	0	0.00
	**HUAVE (26/26)**	11	0.42	13	0.50	2	0.08	19	0.73	7	0.27	0	0.00
	**MAZATECO (59/61)**	4	0.07	21	0.36	34	0.58	56	0.92	4	0.07	1	0.02
	**MIXE (89/88)**	12	0.13	43	0.48	34	0.38	84	0.95	4	0.05	0	0.00
	**MIXTECO (134/133)**	15	0.11	40	0.30	79	0.59	129	0.97	4	0.03	0	0.00
	**ZAPOTECO (66/65)**	4	0.06	19	0.29	43	0.65	65	1.00	0	0.00	0	0.00
	**TOTAL AMERINDIANS (499/500)**	60	0.12	174	0.35	265	0.53	473	0.95	26	0.05	1	0.00
	**MESTIZO (47/39)**	2	0.04	25	0.53	20	0.43	33	0.85	6	0.15	0	0.00
**SOUTH EAST (SE)**	**CHUJ (17/17)**	1	0.06	4	0.24	12	0.71	17	1.00	0	0.00	0	0.00
	**JAKALTEKO (40/40)**	3	0.08	10	0.25	27	0.68	34	0.85	6	0.15	0	0.00
	**KANJOBAL (29/29)**	2	0.07	9	0.31	18	0.62	29	1.00	0	0.00	0	0.00
	**KAQCHIKEL (36/37)**	0	0.00	11	0.31	25	0.69	35	0.95	2	0.05	0	0.00
	**MAM (45/44)**	4	0.09	17	0.38	24	0.53	40	0.91	4	0.09	0	0.00
	**MAYA (234/243)**	49	0.21	112	0.48	73	0.31	198	0.81	45	0.19	0	0.00
	**MOCHO (15/15)**	0	0.00	3	0.20	12	0.80	15	1.00	0	0.00	0	0.00
	**TOJOLABAL (46/45)**	6	0.13	28	0.61	12	0.26	36	0.80	9	0.20	0	0.00
	**TOTAL AMERINDIANS (462/470)**	65	0.14	194	0.42	203	0.44	404	0.86	66	0.14	0	0.00
	**MESTIZO (87/87)**	20	0.23	47	0.54	20	0.23	74	0.85	13	0.15	0	0.00
	**TOTAL AMERINDIANS (1813/1824)**												
	**TOTAL MESTIZO (607/537)**												

The frequency of the 677T allele was clearly enriched in the S region of the country, followed by the CE and SE regions (Fig 1). The 677T allele and TT homozygote frequencies showed the lowest values in MA groups from the N (13–47% and 0–19%, respectively) and in a few groups from the other geographic regions, such as Purepechas (CW), Pames (CE), and Huaves (S) (Table 2 and Fig 1). All other MA groups had very high 677T allele and TT homozygote frequencies, ranging from 55–90% for the T allele and 26–80% for the TT genotype and these frequencies were the greatest in the world. Notably, the highest frequencies of the 677T allele were observed among Mochos (90%), Kaqchiquels (85%), and Chujs (82%), which all belong to Mayan ethnic groups, inhabiting the SE of Mexico (Fig 1). The C677T genotype and allele frequencies of ethnic groups in each geographic region are detailed in Table 2 and Fig 1, respectively.

Of note, the geographic distribution of the 1298C frequency followed a gradient opposite to that observed for C677T frequencies (Fig 2). The Mayan ethnic groups Chuj, Mocho, and Kanjobal, (SE), as well as the Seri (N), Pame (CE), Mazahua (CE), and Zapoteco (S) were monomorphic for the A allele of the A1298C variant. In contrast to the frequency observed for the 677T allele, the 1298C allele was enriched in the N region of the country. The highest frequency was found in the Mayo population (22%), and it was twice the value observed in the two neighboring groups in the N: Tarahumaras (11%) and Yaquis (11%) (Fig 2). Homozygote CC alleles were present in only 0.4% of the MA population and 2% of the MEZ population (Table 1).

**Fig 3:**
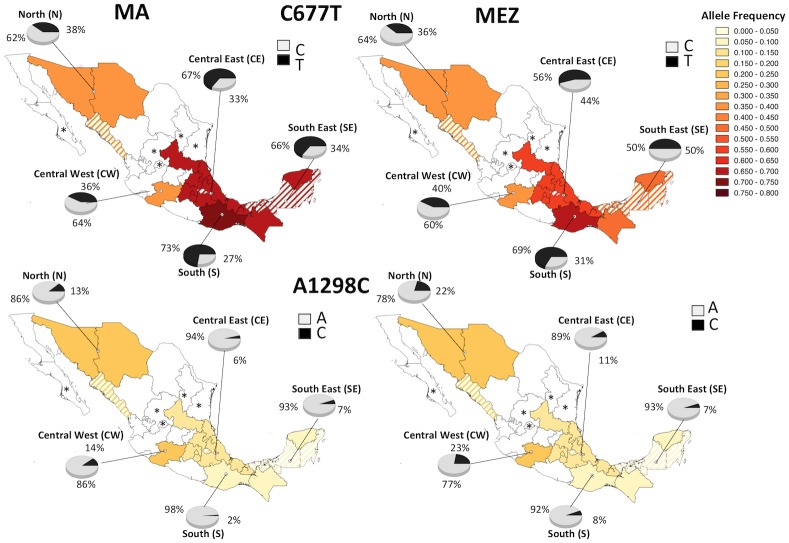
Distributions of the C677T and A1298C variants within each geographic region among the MA and MEZ populations. Striped States were not sampled because they are inhabited by neighboring indigenous included in this study. *States without indigenous population [32].

Since it has been suggested that the Amerindian ancestry of the MEZ genome is mainly from neighboring ethnic groups, we compared the 677T and 1298C allele frequencies between MAs and MEZs by geographical region. The frequency distribution of both *MTHFR* polymorphisms in the MA population was recapitulated in the MEZs of the same region, and like the MAs, the MEZ group showed opposite allele frequency gradients from southern to northern Mexico. The allele frequencies between the MA and MEZ groups were significantly different (*P*≤0.02) in the CE and SE regions for the C677T polymorphism, and in the N, CE, and S for the A1298C variant (Fig 3 and S2 Table). For this analysis, we did not consider the Huaves (S), Seris (N), Pames (CE), and Tojolabales (SE), according to the exclusion criteria mentioned in the Materials and Methods section.

Based on results from the Haploview program, we found that neither of the two variants was in LD (r^2^ values = 0.10 and 0.07, respectively) in either the MA or the MEZ populations. Interestingly, the AT haplotype (A1298C/C677T) was the most frequent in both populations. The CT haplotype was nonexistent in the MA population and the less frequent in the MEZ population. We found no double homozygotes (677TT/1298CC) (S1 Fig).

## Discussion

The Mexican population is mostly Mestizo, a group that is genetically characterized by the admixture of three primary ancestral roots: Amerindian (49 to 65%), European (30 to 45%), and African (3% to 5%) [17, 18]. Additionally, there is a genetic gradient of Amerindian-European mix running from southern to northern part of the country [17, 18]. According to the National Institute of Statistics and Geography (INEGI), 14.9% of the Mexican population is Amerindian, which is one of the largest indigenous populations in the world [19, 20]. Recent studies have reported a clear contribution of Native American alleles to the prevalence of some diseases in Amerindian-derived populations [33, 34]. Among the possible Amerindian genetic contributions for the Mestizo genome are the *MTHFR* variants, which confer a susceptibility to severe phenotypes, like NTDs and other pathologies. The two most common polymorphisms in *MTHFR*, C677T and A1298C, have been associated with reduced enzyme activity. In particular, the 677TT homozygous causes a 50% reduction in enzyme activity [2].

In this study, we showed the frequency of the 677T and 1298C alleles in the largest cohort of MAs included in a genetic study and a sample of MEZs who were geographically-matched. Remarkably, the frequency of the 677T in Mexican individuals and particularly in MA people, was the highest worldwide. In contrast, the frequency of the 1298C risk allele in Mexicans was the lowest in the world. In addition, the frequency of 677T allele showed an increasing gradient from northern to southern Mexico in both populations; while the 1298C allele frequency showed the opposite gradient (Fig 3). This finding demonstrates the great ethnic diversity and heterogeneity of the genetic background in the country. Consistent with previous studies [7, 8, 13, 16], our findings suggest that one of the major contributions of the 677T allele in MEZ was the Indian admixture; while, the 1298C allele is mainly derived from European genomes. Some ethnic groups had the highest frequencies in the country for the 677T allele. Mocho, Kaqchiquel, and Chuj, belonging to the Mayan linguistic family inhabiting the SE region, had frequencies >80%, and Mazateco, Mixteco, Zapoteco, Totonaco, and Mazahua from the S and CE regions had frequencies >70% (Fig 1). Notably, some ethnic groups showed particularities; for example, the Seris (N), Pame (CE), and Huave (S) had lower 677T allele frequencies than other groups that co-inhabited the same regions (13% *vs*. 32–47%; 35% *vs*. 57–74%; and 33% *vs*. 59–81%, respectively) (Fig 1). Also, we found seven ethnic groups that were monomorphic for the A allele of the A1298C polymorphism (Seri, Pame, Chuj, Kanjobal, Mocho, Mazahua, and Zapoteco) (Fig 2). In contrast to the 677T allele, the MA groups from the N (32–47%), with the exception of the Seris, had the highest 1298C allele frequencies (11–22%). These findings may reflect the particular features and history of migration and isolation throughout the centuries of each ethnic group, including the 5 Nahuatl groups, where heterogeneity was also observed among them.

Since there was no difference between 677T and 1298C allele frequencies observed in the newborns of this study (52% and 14%, respectively) and those previously reported in Mestizos adults (44–58% and 14.7%, respectively) [6, 13, 16, 35], we discard that the age differences between MA and MEZ groups could have an effect on the frequencies of both alleles. Although over time, we should be assured that preventive health policies, like the folic acid supplementation campaign, have not had a selection effect to increase the *MTHFR* allele frequencies that could appear in future generations. Later, we compared the allele frequencies of *MTHFR* variants between geographically-matched MA and MEZ individuals distributed throughout the Mexican territory. Seris, Huaves, Pames, and Tojolabales were excluded form this analysis because their *MTHFR* allele frequencies were significantly different from all the MAs inhabiting the same geographical region, these ethnic groups were found to be genetically divergent in previous population genetic studies [18] or they did not contributed to the genetic structure of the MEZ, according to historical data [26–28]. When we compared the 677T and 1298C allele frequencies between both samples by geographical region, the frequency distributions of both *MTHFR* polymorphisms were similar in the MAs and in the MEZs of the same region (Fig 3 and S2 Table). These results agreed with previous genetic studies that reported the Native American ancestry in MEZs is mainly derived from the co-inhabiting indigenous populations, even though the MEZ do not have had strict cultural and geographical barriers, like the MA population [17, 18, 36, 37].

Additionally, we found the highest frequency of the 677T allele in the regions where there is the highest incidence of NTDs in Mexican Mestizos (S, CE, and SE) [19, 35] and interestingly, it was consistent with the regions where Amerindian groups made a major contribution to the Mexican gene pool [17, 18, 36]. Remarkably, most of the studies performed prior to the global folic acid campaign reported an association of the 677T *MTHFR* allele with an increased risk of NTDs [38–41], but this association was not replicated in studies carried out in the post campaign period. These observations suggest that it is possible that the association between the *MTHFR* variants and the NTD anomalies may have been masked when the campaigns for global folic acid supplementation were introduced. Nevertheless, we cannot discard the influence of non-genetic factors on the incidence of NTDs, since they have a complex etiology that comprises not only the influence of genetic factors but also of environmental factors. It has been reported that the consumption of fumonisin-contaminated maize is associated with an increased risk for NTDs in Central Americans and Mexican-Americans living near the Texas-Mexican border [42, 43]. However, although the diets of both MAs and MEZs are based on maize, folic acid supplementation had a similar impact as in countries with high frequencies of the 677T polymorphism and high prevalence of NTDs, but where maize-based diets are non-existent. Otherwise, it was reported that in pregnant LM/Bc mice exposed to fumonisin B1, maternal folate supplementation only partially rescued the NTD phenotype [44], suggesting that the disruption of the folate transport by fumonisins is not fully counteracted by the folic acid supplementation. Unfortunately, in spite that Mexico has one of the highest NTD incidences worldwide, few studies have evaluated genetic and environmental impact on the high incidence of pathologies like NTDs.

In order to understand why particular populations have a high frequency of deleterious alleles, it has been suggested that besides of the evolution, many other aspects such as host-pathogen interactions, dietary adaptation and others, might have a crucial role in positive selection on genetic variants, including those deleterious alleles [45]. As an example, we previously reported that the Mexican population has the highest frequency over the world of an *IRF5* haplotype, which increases the immune innate response. Although this variant appears to be the most important genetic factor conferring risk for systemic lupus erythematosus (SLE) in the Mexican population, it is possible that individuals carrying it would have a genetic advantage when fighting infectious diseases [34]. Accordingly, Fodil-Cornu et al. reported that *mthfr*-deficient mice infected with murine cytomegalovirus have a more efficient viral replication than their heterozygous or wild-type littermates [46]. The Native Mexican population has been exposed to several periods of adaptations, but it is accepted that its greatest population bottleneck was during the European colonization [47], when some new infectious diseases such as influenza, smallpox and measles were introduced to the indigenous population. Indeed, the highest frequency of 677T was found in those Mexican indigenous groups from the Mesoamerican region, where the Spanish colonization had the major influence. Thus, it is possible that the *MTHFR* 677T allele was part of the set of variants that conferred a survival advantage to the Amerindian-derived population, similar to those described for the *IRF5* haplotype [34].

In conclusion, the high frequency of the 677T allele found in indigenous people suggests that the Amerindian ancestry of the Mexican population contributes to the high prevalence for diseases associated to folate deficiency, such as NTDs. It is tempting to believe that NTDs have reached a high prevalence in Mexico because the Amerindian heritage of alleles provides a survival advantage, similar to those reported for other alleles like the SLE risk haplotype in *IRF5*. Further detailed genetic and environmental analysis should be performed in order to elucidate the impact of the *MTHFR* polymorphisms on the high incidence of severe pathologies like NTDs.

## Supporting Information

S1 Fig *MTHFR* haplotype frequencies in MA (A) and MEZ (B). LD r2 value = 0.10 and 0.07, respectively.
(TIFF)

S1 Table Comparison of MTHFR 677T and 1298C allele frequencies among the different populations, including those in the present study, MAs and MEZs. (DOCX)

S2 Table Comparison of MTHFR 677T and 1298C allele frequencies between MA and MEZ matched by geographical region. (DOCX)
