# Supplementary figures and images for: Heterogenous Distribution of MTHFR Gene Variants among Mestizos and Diverse Amerindian Groups from Mexico
Source: PLoS One. 2016 Sep 20;11(9):e0163248. doi: 10.1371/journal.pone.0163248 (PMC5029802; doi:10.1371/journal.pone.0163248)

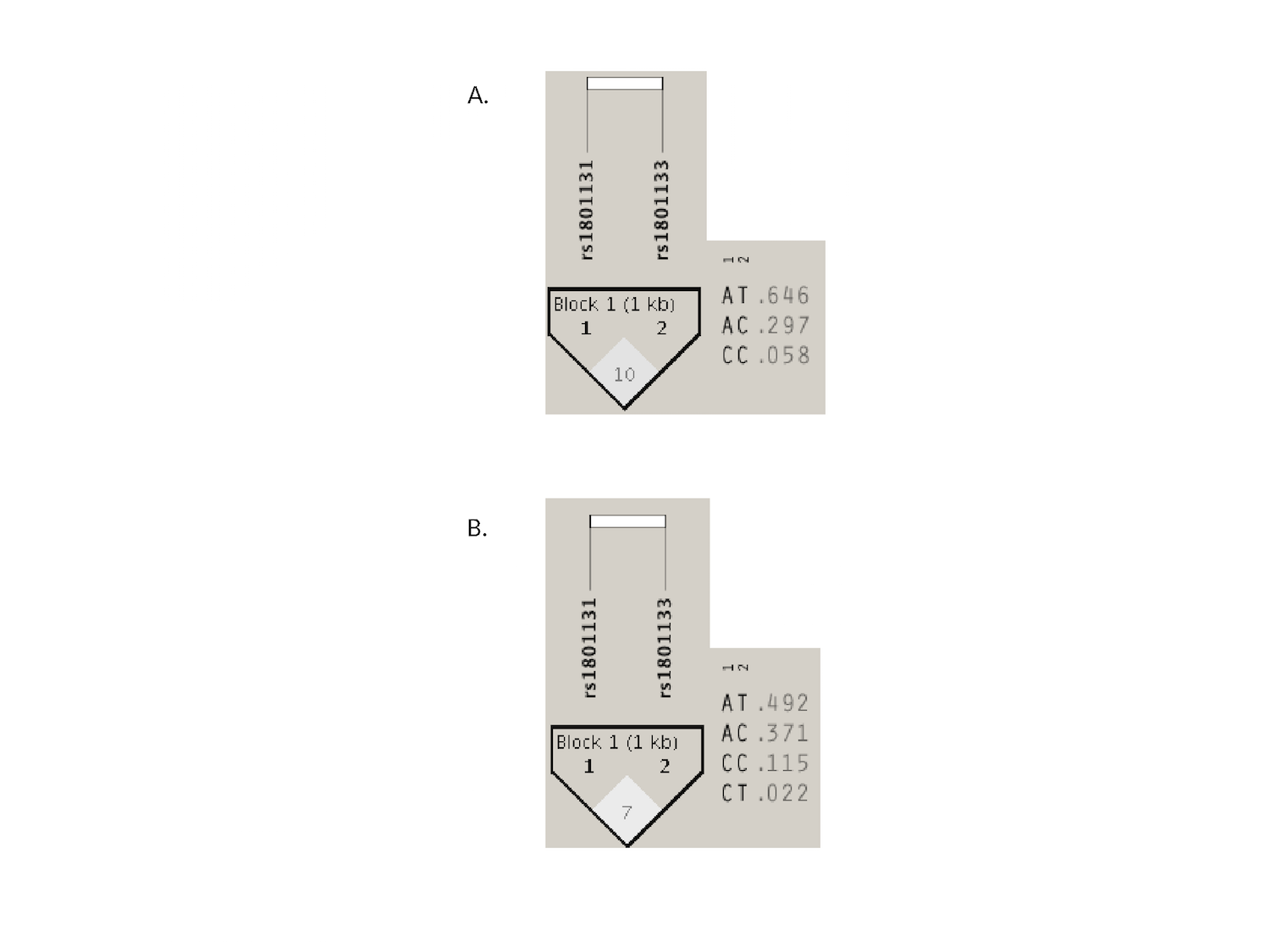

Supplement: S1 Fig — LD r2 value = 0.10 and 0.07, respectively. (TIFF) [file pone.0163248.s001.tiff]
