# Supplementary material for: Heterogenous Distribution of MTHFR Gene Variants among Mestizos and Diverse Amerindian Groups from Mexico
Source: PLoS One. 2016 Sep 20;11(9):e0163248. doi: 10.1371/journal.pone.0163248 (PMC5029802; doi:10.1371/journal.pone.0163248)
Supplement: S2 Table — (DOCX) [file pone.0163248.s003.docx]

**S2 Table.** Comparison of *MTHFR* 677T and 1298C allele frequencies between MA and MEZ matched by geographical region.
